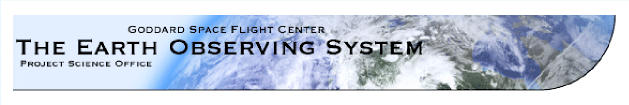# EHPnet: The Earth Observing System

**Published:** 2005-02

**Authors:** Erin E. Dooley

In 1991, the National Aeronautics and Space Administration (NASA) embarked upon the extensive and comprehensive Earth Science Enterprise, a program to help us better understand what is happening on the Earth’s surface. The centerpiece of the Earth Science Enterprise is known as The Earth Observing System (EOS). The EOS website, located at **http://eospso.gsfc.nasa.gov/**, is designed to keep scientists, educators, and the news media informed about the system and its activities.

EOS is composed of a series of satellites conducting long-term global observations of the land surface, biosphere, solid Earth, atmosphere, and oceans. The first EOS satellite, Terra, was launched into orbit in December 1999. NASA hopes the data gathered by the system, the first to provide integrated measurements of processes occurring on Earth, can be used to help better predict climate change, oversee conservation efforts, and improve forecasting to help farmers, fishers, and other weather-dependent tradespeople.

The For Scientists section of the website provides links to overviews of the field experiments that EOS conducts, and contains information on what sorts of measurements EOS takes and what types of instruments are used. Also within this section are links to brochures, reference books, data product catalogs, a bibliography of EOS-related publications, and a calendar of workshops and conference seminars conducted by EOS personnel.

Teachers looking for EOS-related resources can visit the For Educators section. The EOS Education Project, with a link located under the Educational Links subhead, has been developed for teachers and students at all levels to teach about topics including geographic information systems and remote sensing. The project offers outreach programs for teachers and Internet-based classes. This section also features a directory of other EOS-related teaching materials, including brochures, fact sheets, posters, lithographs, and lesson plans that can be downloaded or ordered for free. Also available are online tools for calculating when a satellite will pass overhead and tracking various satellites’ current location in the sky. Links to other websites include the main NASA Earth Science Enterprise educational website and the homepages of related agencies such as the National Science Foundation.

News writers have their own section on the website. Available under For News Media is the Earth Observatory Newsroom, which contains press releases on the latest EOS research, NASA news announcements, and a list of newly published research. This section also features the EOS Global Change Media Directory 2001. This resource lists 343 scientists working in fields such as ozone chemistry, global warming, and ecosystems who have expressed interest in working with the media. Science writers’ guides to individual spacecraft provide extensive background material and contact information.

The site’s Data Services section provides links to the different types of data that the EOS and its partners provide. This section links to NASA’s Global Change Master Directory website, which provides extensive data sets on 13 topics, including agriculture, human dimensions, oceans, and sun–Earth interactions. Subscribers can add to the data sets and participate in an online discussion list.

## Figures and Tables

**Figure:**